# Digital pathology – Rising to the challenge

**DOI:** 10.3389/fmed.2022.888896

**Published:** 2022-07-22

**Authors:** Heather Dawson

**Affiliations:** Institute of Pathology, University of Bern, Bern, Switzerland

**Keywords:** digital pathology, scanner acquisition, validation, image analysis, artificial intelligence

## Abstract

Digital pathology has gone through considerable technical advances during the past few years and certain aspects of digital diagnostics have been widely and swiftly adopted in many centers, catalyzed by the COVID-19 pandemic. However, analysis of requirements, careful planning, and structured implementation should to be considered in order to reap the full benefits of a digital workflow. The aim of this review is to provide a practical, concise and hands-on summary of issues relevant to implementing and developing digital diagnostics in the pathology laboratory. These include important initial considerations, possible approaches to overcome common challenges, potential diagnostic pitfalls, validation and regulatory issues and an introduction to the emerging field of image analysis in routine.

## Introduction

In many parts of the world, at least part of pathologists’ work has become “digital,” i.e., conventional routine replaced by digital images. However, many pathology institutes that pursue the path to digital pathology realize that this is not always as straightforward as it seems. As technical aspects and possibilities advance at nearly dizzying speed, it must be emphasized that a digital approach will affect many fundamental aspects of daily routine in a pathology lab. Careful planning and certain practical considerations can be paramount for a smooth transition to an efficient digital workflow. The objective of this article is to provide a broad overview of digital pathology for the general pathologist and address real-world issues which may be underrepresented in the literature. Select review articles, white papers and consensus guidelines are recommended for further in-depth reading (1–5).

What constitutes digital pathology? Broadly interpreted, this can entail any sort of work involving a digital image. For instance, digital photography to capture and store macroscopic images can be considered one of the first widespread aspects of digital pathology. Real-time sharing of pathology images with a common viewer (telepathology) has been favored for some time by hospital systems with multiple sites and increasingly implemented during the COVID-19 pandemic (6). The most complex application is the digitization and storage of whole slides images (WSI), either for use in diagnostic routine or for educational or research purposes.

## Advantages and challenges of digital pathology

There are various potential advantages of digital pathology, including:

- Patient safety-related: Provided storage is reliable and data secure, a scanned slide cannot be lost or broken. Most viewer programs also have annotation functions, which enable the documentation of exact measurements (for instance, the exact infiltration depth of a tumor or distance to resection margins).
- Access to slides: One of the most obvious drivers of digital pathology in the past 2 years has been pandemic-related to enable remote work. The retrieval of archived slides can also be considerably simplified and does not require staff assistance. Depending on the laboratory setup, scanned cases can also improve workflow by reducing physical slide distribution.
- Sharing cases: Digitized scans can be easily shared for second opinions, interdisciplinary tumor conferences and educating students and residents.
- Research: Established archives of scanned WSI or tissue microarrays can be mined for research projects and enables centralized, standardized repositories of research data (including patient data, tissue-related data etc.).
- Implementation of automated algorithms: WSI are prerequisite for the vast possibilities of additional image analysis software for diagnostic assistance (see below).

## Initial considerations

The benefits of digital are certainly met with their share of challenges, both from a technical and organizational point of view. First, it should be clear that there is no “one-size-fits-all” solution and that needs of individual institutions will differ. Prior to making large investments, current system requirements should be specified and future ones anticipated. Important considerations may be:

- Scope of application: Is the aim to go fully digital or only scan a certain amount of slides? The amount of expected slides to be scanned and the scanning speed will influence the type and number of scanners to purchase.
- What are system requirements and IT resources? How can storage space be ensured and how much is needed? How can graphics processing units/central processing units (GPUs/CPUs) be accessed? This will highly depend on the planned scope of application. For instance, assuming a biopsy slide output of 1,600/day and an average of 2 GB/slide, 1 PB/year should be calculated if all scans are to be archived.
- System compatibility: How is the interface with the laboratory information system (LIS) and how compatible is the scanner with barcodes from different manufacturers?
- Requirements for access to scans and image management system (IMS): Should scans be available *via* web browser? How can slides be shared? What are requirements for annotation and measurement tools? How can patient metadata from the LIS be integrated to the IMS?
- Workflow integration: How does scanning harmonize with other lab processes? Is continuous loading/unloading possible?
- Openness of system for application of plugin software: Does the scan and proposed IMS enable the use of desired third party software?
- Regional differences in regulatory issues: Is FDA or equivalent approval required for a scanner to be used in a diagnostic setting?
- Financial aspects: Transitioning to a digital pathology workflow requires considerable short-term investments and additional personnel. However, well-planned implementation may result in increased productivity in the long-term, and potentially counteract shortages in trained pathologists who are faced with an increased case load (5, 7). The business case and potential financial gains of a particular pathology institute highly depend on the scope of the project and pre-existing efficiency of the laboratory setup (7).

Some points can only be adequately addressed in a trial period. At the beginning, scanner acquisition is typically one of the first large investments in implementing digital pathology. For instance, a possible approach would be to test several scanners and submit them to a performance test (Table 1), including (8):

**TABLE 1:** Example performance test for scanners under consideration for purchase.

Technical aspects	
File size	File sizes can vary considerably (up to over fourfold) among different scanners and will therefore have a major impact on the storage space needed.
Scan time at 40× magnification and for dayload	The scanning speed can be a bottleneck in the diagnostic workflow depending on the number of slides, the type of specimen (biopsies vs. resections) and the number of pathologists to be signing out cases digitally. In our experience, scan times may vary up to a minute per slide on different scanners at the same magnification.
Interruptions	The vulnerability to interruptions is one of the most important aspects of scanner performance and can have an especially profound impact on overnight scanning. Software, hardware and slide-related issues may all contribute to interruptions.
Rescan rate	After quality control check, establish the proportion of slides that need to be rescanned (e.g., due to focus issues and missing tissue).
Focus	Tissue can be either entirely or only partially out of focus. The technology of continuous autofocusing can result in different areas of the slide being out of focus but lower rates of WSI completely out of focus in comparison to scanners using focus points.
Tissue identification	Scanners use their own algorithms to detect tissue on a slide and therefore variability in tissue detection may be seen among different scanners. For patient safety reasons it is crucial that all tissue is scanned and this must be ensured for each slide. Faintly stained tissue (e.g., myxoid substance or fat) can be missed and only partially scanned by some scanners.
Openness of file format	The scan format should ideally be open for the integration for an independent choice of image analysis tools and convertible into other formats.
**Workflow-related aspects**	
User friendliness	The laboratory staff should be involved in evaluating the usability of scanner software and hardware.
Loading of racks	There are several possibilities: manual loading of slides one by one, overturning of racks from staining machines in one step and direct loading of racks from stainers. However, slides with wet mounting medium tend to stick to the rack and will not be scanned.
Continuous scanning	Some scanners stop to open, others continue with the process even if opened to reload, which can have a considerable effect on case distribution depending on the workflow.
LIS integration	This is crucial, especially for work in the remote setting in terms of work lists and case management. Cooperation with LIS providers is important to ensure that the requirements of the institute are met.
**MD-related aspects**	
IMS	The IMS may be from a different provider than the scanner itself, but is essential for digital sign-out and should be considered during scanner testing. Pathologists should feel comfortable using the IMS, which should provide certain tools (measurement, area calculation, regions of interest, snapshot etc.).
Pathologist evaluation	Pathologists should compare their impression of WSI in terms of quality of the scans and their level of diagnostic confidence. A case set should including potentially difficult cases (e.g., special stains containing microorganisms). Side by side comparisons of WSI from different can be especially helpful in the decision making process.

- Technical aspects: Speed, file size, interruptions, rescan rates, focus and tissue identification issues; what is the proportion of poor quality scans (blurriness, incomplete scans).
- Workflow-related aspects: Handling and user-friendliness, continuous scanning, LIS integration, openness of file format.
- Medical aspects: How do MDs rate the quality of scans including special stains and the usability of the viewer? What is the general impression among different scanners?

## Getting started

The implementation of digital pathology is complex and interdisciplinary. Key players include IT, laboratory staff, MDs and institute management. Although the strategic decision to use digital pathology is traditionally made at the management level, it is important to involve all units at an early stage of development. Our digital pathology team includes a project manager, 2 pathologists, a specialized technician, an IT specialist responsible for our LIS and a LEAN specialist, and meets regularly with the head of the pathology lab and representatives of our LIS and IMS providers. The digital pathology group also works closely with our computational pathology researchers. However, the main goal is the continuous development of digital pathology for diagnostic routine in the department.

**FIGURE 1:**
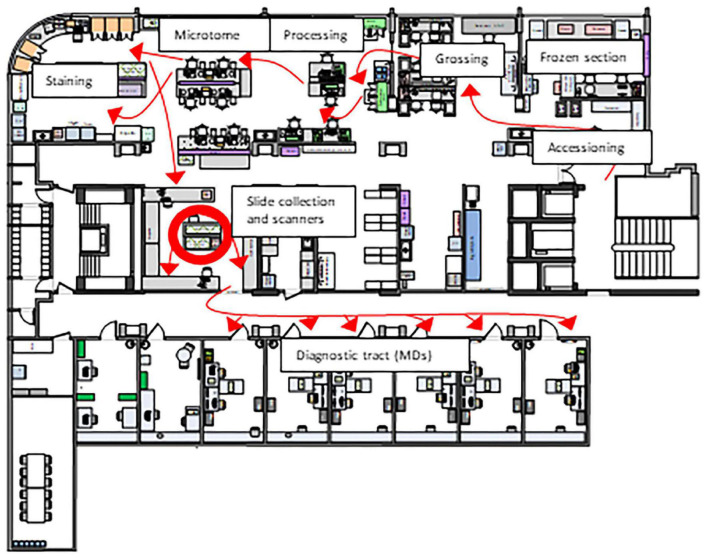
Layout of our histology laboratory according to Lean principles. The red arrows that represent the path taken by a specimen from acquisition to the MD are unidirectional (“LEAN biopsy/resection street”). Therefore, the Lean solution was to place scanners in the slide sorting area (red circle), which minimizes waste both for a hybrid solution (digital and conventional sign-out) and fully digital sign-out.

Our histology lab is organized according to Lean principles (9). As many processes in the lab including the physical transfer of histology slides already revolve around a Lean workflow we were faced with the challenge of integrating an additional step. Since scanning needs to match the batch-based distribution of slides, continuous scanning is necessary to keep our workflow running. Also, space restrictions and an ideal position of the scanner need to be taken into consideration (Figure 1). However, a Lean approach to digitizing cases is not just limited to the scanner itself. Automated processes starting with accession can help to minimize waste and save time. For instance, if correctly identified, a particular case can be processed so that slides are scanned and algorithms already run before the scan reaches the pathologist. In addition, standardized sampling and measurements of gross specimens enable the automated labeling of scanned slides and necessary elements directly transferred to synoptic reports.

Some practical aspects of tissue processing will influence digitizing slides. For example, small biopsies very close to the edges of the slide may not be identified by the scanner, which can lead to relevant patient safety issues (see discussion on quality control). Therefore, this must be taken into account at the microtome. Also, some scanners will accept racks directly from some stainers, but it must be ensured that slides are dry, as slides with wet mounting medium tend to stick to the rack and will not be scanned. The use of barcodes for specimen labeling is used in many laboratories and is required for a digital workflow. Regardless of whether slides are scanned or not, barcodes are the basis of a digital tracking system, which can drastically improve patient safety and also be used as a management tool (e.g., measurement of turnaround times and performance indicators). Robust barcode reading is also essential for the scanning process and there are several types of barcode printers which vary in terms of price, print quality and scanner readability (10). The highest quality barcodes are produced by thermal printers which are more expensive than other types of printers, but may save time and effort by drastically reducing failure rates in barcode reading. Alternatively, training laboratory staff to recognize poor quality barcodes may also yield acceptable barcode scanning rates.

## The MD team

Prior to routine scanning in our department (also before the COVID-19 pandemic), MDs were involved in rating of several tested scanners in terms of handling issues, comparison of side by side scans and scan quality and asked about their general attitude toward digital pathology in general. Most pathologists were open to the digital future, for instance 75% felt they would be able to make diagnoses on scanned images (8). This is in concordance to a national survey performed in Switzerland in 2019, where the majority of pathologists (66%) also reported feeling comfortable rendering primary diagnoses on scanned slides (11). Inevitably, the importance of digital pathology has risen significantly, leading to increased use digital pathology in daily routine. Unsurprisingly, a more recent poll of Swiss pathologists at the height of the pandemic revealed the use of digital pathology for primary routine diagnostics had more than doubled since onset of the pandemic (30 vs. 13.5%) (12).

In the midst of the rise in digital pathology, it cannot be forgotten that currently, the vast majority of pathologists have completed their training and accumulated years if not decades of experience using conventional light microscopy. Therefore, most pathologists still currently consider conventional microscopy as gold standard not only when it comes to diagnostic confidence, but also speed and efficiency in routine. In our experience, when faced with a high diagnostic workload and the option between digital and conventional work, many pathologists will still prefer to work with glass slides. The optimization of digital pathology in this regard is truly challenging on many levels and should not be underestimated. Even if performance issues as frequently encountered in the setting of remote work can be eliminated, each extra mouse click will add up to a considerable and noticeable amount of extra time spent on sign-out over the course of a busy workday. Investing time to achieve optimal user interface in the LIS and IMS are especially important and require close cooperation between the MDs and technical team.

The perceived ease of digital pathology is an important factor for everyday use in diagnostic routine. Speed, accuracy, ease of use and ergonomics should all be taken into consideration. However, although crucial from a practical point of view, reports in the literature on such aspects of digital sign-out are rare. Certain input devices such as a conventional mouse may be straining, especially when using the wheel for zooming over a prolonged period of time. Here, other types of devices may be preferred, especially in terms of comfort (13).

**FIGURE 2:**
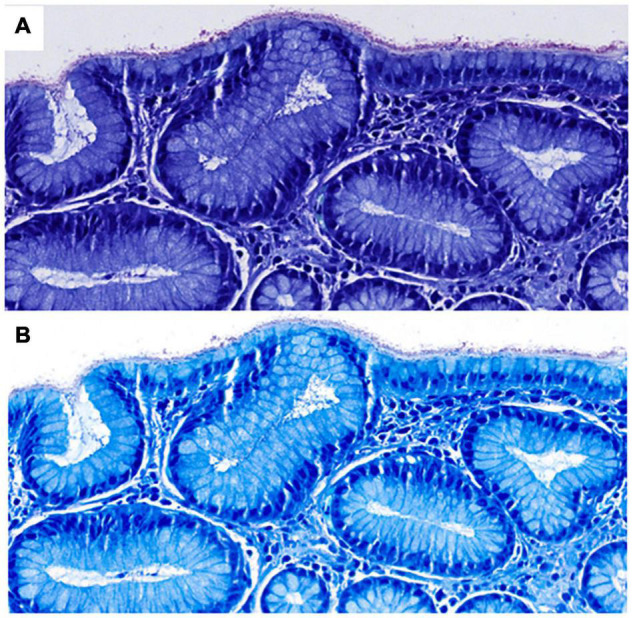
Scanned Giemsa stains from two different scanners **(A,B)** used in diagnostic routine at our institute in a case of H. pylori gastritis (both at 40×, images courtesy of Ursina Begré). Note the differences in color and brightness between scanners. In a survey of our MDs, scanner B provided a higher level of diagnostic security on Giemsa scans of gastric biopsies but both scanners performed similarly at 40×. Therefore, Giemsa stains for gastric biopsies are scanned at higher magnification per default.

Although several studies have demonstrated that digital pathology is non-inferior to conventional light microscopy in a wide spectrum of diseases and organ systems (14, 15), there are still some potential issues to be aware of. For instance, the detection of microorganisms and subtle morphological features such as nuclear details, including mitotic figures and chromatin patterns on scanned slides can be challenging. This can be due to several reasons. For instance, poor quality of scans can be a major source of uncertainty and discrepancies from glass slide diagnoses, in which case rescanning at a higher magnification or Z-stacking may increase diagnostic confidence (Figure 2) (14). However, it has also been suggested that many discrepancies are not due to limitations of diagnosis on WSI as a process but other factors, such as naturally occurring interobserver variability, inadequate tissue processing or lack of experience of the pathologist (16).

## Validation

As digital diagnostics represents a technique that significantly differs from conventional light microscopy, validation tests must encompass the entire digital system to ensure necessary standards are met. This includes the scanning process, the LIS and IMS interface and archiving of WSI. Good practice statements and recommendations of the College of American pathologists (CAP) (3) from which general principles have been adapted by most societies are summarized in Table 2.

**TABLE 2:** Summary of good practice statements (GPS) of the College of American pathologists (3).

GPS 1: All pathology laboratories implementing digital pathology for diagnostic purposes should carry out their own validation studies.
GPS 2: Validation should be appropriate for and applicable to the intended clinical use and clinical setting of the particular application. Validation of WSI systems should involve specimen preparation types relevant to intended use. If a new application for WSI is desired and differs materially from the previously validated use, a separate validation for the new application should be performed.
GPS 3: Validation should closely simulate the real-world clinical environment in which the technology will be used.
GPS 4: Validation should encompass the entire WSI system. However, it is not necessary to validate each individual component (i.e., computer hardware, monitor, network, scanner) of the system or the individual steps of the digital imaging process.
GPS 5: Laboratories should have procedures in place to address changes to the digitized system that could impact clinical results.
GPS 6: Pathologists adequately trained to use the WSI system must be involved in the validation process.
GPS 7: The validation process should confirm all material on a glass slide to be scanned is included in the digital image.
GPS 8: Documentation should be maintained recording the method, measurements, and final approval of validation for the WSI system to be used in the laboratory.
GPS 9: Pathologists should review slides in a validation set in random order. This applies both to the review modality (glass slides or digital) and the order in which slides are reviewed within each modality.

Pathologists themselves are a crucial part of the validation process, especially since not all pathologists may be confident with working in a digital process and should be given the possibility to acquire skills outside live reporting. There are several published recommendations for pathologist validation which are more or less extensive (3, 17, 18) but share the following principles:

- Training and validation cases should reflect real life work and include all types of stains used in diagnostic routine (including special stains and immunohistochemistry).
- Validation should include a comparison between diagnoses made by the same pathologist on glass slides and WSI after a washout period to minimize recall bias and eliminate interobserver variability. All pathologists using digital diagnostics in routine must complete the validation study.
- Documentation of the validation protocol including concordance and discordance of cases. The level of concordance between diagnoses on glass slides and WSI is generally expected to be over 95%.

In addition to diagnostic routine on WSI, all image analysis tools must be validated by each institution prior to clinical use, irrespectively of FDA- (or equivalent) approval status. As specific guidelines for validation studies and acceptance criteria for such tools are lacking, the approval of a validation study design is left up to the discretion of the medical director. However, the basic aspects as mentioned above should be applied: the validation study should be applicable to the intended clinical use in which the tool will be employed and encompass the spectrum and complexity expected to be encountered in diagnostic routine. Important considerations for in-house algorithm validation study design are listed in Table 3.

**TABLE 3:** Considerations for validation study design for image analysis tools.

Ground truth definition	The algorithm output must be compared to a ground truth to establish precision and recall (precision addresses the proportion of positive identifications that was actually correct, i.e., a model with no false positives has a precision of 1.0; recall addresses the proportion of actual positives that were correctly identified, i.e., a model with no false negatives has a recall of 1.0) This can be done in several ways: 1. Manual annotation: most exact method for comparison to algorithm output but time-consuming 2. Eyeballing (if applicable): Region of interest can be pre-set, replicates real-life diagnostic setting 3. Comparison with previously reported values (derived from LIS): Least exact method, region of interest not standardized, but least time-consuming
Case selection	There are no published guidelines on the number of cases that should be included but the case mix should reflect the real life setting in terms of morphological heterogeneity and complexity (e.g., different histological variants/subtypes)
Acceptable range of output values	Define acceptable range of deviation to ground truth. This may depend on clinically relevant cutoffs that determine therapy (e.g., PD-L1, Ki67)
Possible confounding effects	If several scanners are used to run the algorithm on check whether the scanner has an effect on algorithm output
Identify discrepant cases and analyze reasons for discrepancy	Output values outside the defined acceptable range are discrepant to the ground truth. Can systematic reasons be identified (for example threshold of color detection, falsely identified tumor cells etc.)? Are the ground truth values really correct? In the case of substantial discrepancies, support of the provider may be warranted.

## Quality control of whole slides images

Suboptimal WSI can be due either to issues in tissue handling (e.g., folds, tears, poor staining, thick cuts etc.) or the scanning process (focus issues, tissue recognition, etc.). Quality control of WSI is important for troubleshooting and fixing the root of the cause. If left up to the pathologist to identify poor images, important time is lost in which the underlying problem could already have been solved. Therefore, quality control should ideally be performed in the pathology laboratory. Quality control of glass slides may be acceptable on random samples and certain issues picked up by the naked eye of experienced technicians, but quality control of WSI may require opening each scan, which is quite time-consuming and not feasible for many laboratories – especially considering the amount of extra work scanning may entail. Therefore, automated quality control tools are highly desirable to relieve the laboratory staff of this process. Several automated tools have been proposed, some of which merely identify focus quality (19, 20). However, a more comprehensive solution which addresses both laboratory- and scan-related quality control issues would be ideal, especially in a workflow where all slides are digitized. In 2019, the free open-source software HistoQC was introduced as potential tool for automated quality control (21). Although more of a prototype in its initial form, hampered by its complex configuration and limitations in interpreting calculated metrics (22), further developments may be expected for this tool to be used as a ready-to-use program for clinical application in the future.

## Image analysis – friend or foe?

One of the most obvious advantages of digital pathology is the implementation of automated algorithms to assist in diagnostics. Most tools rely on artificial intelligence, which is an umbrella term that encompasses machine learning and deep learning. Machine learning is a term for computer systems that learn and adapt without following explicit instructions by using algorithms and statistical models to analyze and draw interferences from patterns with data. Deep learning is a type of machine learning based on artificial neural networks, which are inspired by the understanding of biological neural networks (23). Image analysis tasks cover a wide range of potential applications. Basic tasks that might already be in use include quantitative analyses such as a count of objects (e.g., mitoses, tumor-infiltrating lymphocytes, immunohistochemical biomarkers like Ki-67 in breast cancer etc.) that are traditionally perceived as repetitive and time-consuming in routine and may be prone to inter- and intraobserver variability (24).

Other applications may focus on evaluating diagnostic features, such as distinguishing diseased from normal tissue, grading cancers or differentiating between different cancer types. Some systems appear highly successful in tumor detection with reported near perfect accuracy rates (25). There are currently two approaches to implementing cancer-detection algorithms, either as a “first read,” in which slides are analyzed prior to the pathologist, highlighting suspicious areas with the aim to improve diagnostic efficiency. In the “second read” approach, slides are evaluated in parallel to the pathologist and notifies in case of clinically relevant discrepancies (for instance a missed focus of cancer tissue) with the intention to minimize error rates. At present, FDA-approval for use in primary diagnostics is limited to only very few products (26) but will certainly be expanded in the near future.

More advanced applications include predictions that might not be evident to the human eye. Comprehensive image analysis for complex aspects such as different cell types in the tumor microenvironment or tumor heterogeneity is simply not feasible without some kind of computational method. Clinically relevant information such as survival, molecular classifications or response to therapy can possibly be predicted by a WSI. For instance, a published algorithm reported predicting HER2 amplification in breast cancer with an AUC of 0.70 (95% CI 0.63–0.77) and more favorable survival in trastuzumab-treated patients according to the automated prediction status (ERBB2 score) (27). In addition, a high ERBB2 score in CISH-HER2 negative patients was associated with unfavorable survival, indicating that the algorithm picked up HER2-cancer-like morphological features linked to poorer outcomes. Another potential tool aiming for a one-stop-shop workflow for pan-cancer image-based detection of clinically actionable genetic alterations based on H&E stained slides was able to significantly predict mutational status of at least some oncogenic genes in 14/14 cancer types, whereas the highest accuracy was achieved for lung, colorectal and breast cancer (AUC 0.60–0.78; 0.65–0.76 and 0.66–0.78, respectively) (28). Evidently, such tools are still of exploratory nature, but it can be assumed that they too will be used in the clinical setting at some point. However, several issues concerning validation in algorithms that could directly influence clinical decision-making will need to be addressed – especially if artificial intelligence (AI)-tools are considered to replace the current gold standard of molecular-based assays - underlining the need for investigation in randomized clinical trials.

Although many studies report high accuracy rates of AI algorithms, this does not necessarily translate to usability in routine (29). Algorithms themselves should undergo rigorous validation on multiple levels according to good practice guidelines (23), ensuring exposure to a wide variety of data sources (including external validation sets, different scanners etc.). However, even if all measures are taken to meet these standards, real-life data will still typically have many more sources of nuances and variation than the datasets used for training and validation. Additionally, it must be emphasized that algorithms are far from replacing the work of pathologists, which is to take a integrative approach incorporating medical knowledge, diagnostic experience and the particular circumstances of a certain case to either make a final diagnosis or decide if further work-up is needed. Therefore, for the foreseeable future a constructive strategy could be to view AI tools as a way of increasing efficiency and enhancing the quality of diagnoses in routine (30).

## Globally relevant issues

Clearly, digital pathology requires significant financial investments that might be affordable for high-income countries but exorbitant for many low-and middle-income countries. Although listed prices vary greatly depending on the product, a single high-throughput scanner will cost between $100’000 to $400’000 (7). Further costs include hardware (high-resolution display screens and high-performance computers), additional software, IT infrastructure and personnel. In addition to financial issues, low-and middle-income countries tend to suffer from a general shortage of pathologists which often prefer to practice in urban agglomerations, whereas a large proportion of the population lives in rural areas (31). Therefore, unfortunately, the wealth of a particular region will influence potential applications of digital pathology. For instance, especially developing countries can benefit from educational aspects of digital pathology with increasing availability of virtual or hybrid courses, eliminating travel costs. Also, telepathology can aid in seeking second opinions from colleagues, increasing diagnostic quality. Algorithm-assisted diagnostics can also potentially be used to provide some relief to a high workload. Although traditionally, all of these aspects require some financial investment in terms of scanning machines, recent efforts have been made to develop a cost-efficient real-time microscope-based solution for deploying AI tools, eliminating the need for WSI (32).

## Conclusion

Digital pathology is a complex endeavor which requires careful planning in advance. The preparation phase should include detailed analysis of the requirements, starting point and goals of a particular institute as there is no one-size-fits-all solution. In our experience, it is important to assemble a team from the beginning consisting of key players involving all areas which will be affected by digital pathology (including MDs, lab technicians, researchers, etc.). Test phases with structured performance analysis are recommended prior to purchasing large equipment such as scanners. To ensure equivalent diagnostic quality to conventional routine diagnostics, validation studies on the process of digital pathology as a whole and add-ons such as image analysis tools must be performed by each laboratory according to guidelines or good practice standards. Acceptance of digital pathology generally appears to be high, although affinity for digital work can vary among pathologists. Providing a user friendly, simple and ergonomic digital workspace requires effort but can ease the transition to routine digital diagnostics. At the moment, AI algorithms can be viewed as a possibility to aid and enhance certain aspects of routine diagnostic work rather than replacing the pathologist. Combining forces of human experience, research and AI is bound to help advance an efficient workflow and personalized healthcare treatment.

## Author contributions

The author confirms being the sole contributor of this work and has approved it for publication.

## Conflict of interest

The author declares that the research was conducted in the absence of any commercial or financial relationships that could be construed as a potential conflict of interest.

## Publisher’s note

All claims expressed in this article are solely those of the authors and do not necessarily represent those of their affiliated organizations, or those of the publisher, the editors and the reviewers. Any product that may be evaluated in this article, or claim that may be made by its manufacturer, is not guaranteed or endorsed by the publisher.
