# Erratum: Digital pathology – Rising to the challenge

**DOI:** 10.3389/fmed.2023.1180693

**Published:** 2023-03-15

**Authors:** Frontiers Production Office

**Affiliations:** Frontiers Media SA, Lausanne, Switzerland

**Keywords:** digital pathology, scanner acquisition, validation, image analysis, artificial intelligence

An omission to the funding section of the original article was made in error. The following sentence has been added: “Open access funding was provided by the University of Bern.”

The original article has been updated.

